# Doe diligence: A regional analysis of antlerless deer harvest regulations in the Midwestern United States of America

**DOI:** 10.1371/journal.pone.0324708

**Published:** 2025-06-04

**Authors:** John P. Draper, Ellen E. Brandell, Jason Isabelle, Chris Jacques, Clint McCoy, Eric Michel, Daniel J. Storm, Caitlin Ott-Conn, Beth Wojcik, Wendy C. Turner, Daniel P. Walsh

**Affiliations:** 1 Wisconsin Cooperative Wildlife Research Unit, Department of Forest and Wildlife Ecology, University of Wisconsin–Madison, Madison, Wisconsin, United States of America; 2 Colorado Parks and Wildlife, Fort Collins, Colorado, United States of America; 3 Missouri Department of Conservation, Columbia, Missouri, United States of America; 4 Illinois Department of Natural Resources, Lena, Illinois, United States of America; 5 Ohio Department of Natural Resources, Columbus, Ohio, United States of America; 6 Department of Wildlife, Fisheries and Aquaculture, Mississippi State University, Starkville, Mississippi, United States of America; 7 Wisconsin Department of Natural Resources, Eau Claire, Wisconsin, United States of America; 8 Michigan Department of Natural Resources, Wildlife Division, Marquette, Michigan, United States of America; 9 Wisconsin Department of Natural Resources, Madison, Wisconsin, United States of America; 10 U.S. Geological Survey, Wisconsin Cooperative Wildlife Research Unit, Department of Forest and Wildlife Ecology, University of Wisconsin–Madison, Madison, Wisconsin, United States of America; 11 U.S. Geological Survey, Montana Cooperative Wildlife Research Unit, Wildlife Biology Program, University of Montana, Missoula, Montana, United States of America; Bowling Green State University, UNITED STATES OF AMERICA

## Abstract

Wildlife management in the United States of America (US) is primarily delegated to the individual states wherein state wildlife agencies manage wildlife populations to achieve multiple and sometimes conflicting objectives. White-tailed deer (*Odocoileus virginianus*) are an important species in the Midwestern US whose populations are primarily managed through recreational hunting. Managers aim to adjust populations by altering the harvest of antlerless (usually female) animals by changing the number of harvest permits available, hunting season lengths, or applying incentive programs like earn-a-buck, where a hunter must harvest an antlerless deer before they may harvest an antlered deer. We estimated the effect on antlerless deer harvest from changes in these regulations and changes in the number of licensed hunters across eight states in the Midwest. We used a Bayesian hierarchical model to estimate individual state and regional (i.e., across all states) effects. We found that increasing antlerless harvest permits increased antlerless harvest; however, this effect plateaued as the number of available permits increased. Providing unlimited harvest permits increased harvest, but the same increases were achieved by minimally increasing the number of limited harvest permits. Increasing the length of hunting season had a generally positive effect on antlerless harvest but the effect was non-linear and state dependent. The earn-a-buck incentive program resulted in the largest estimated increase in harvest. Finally, the number of licensed deer hunters in a state had a strong positive effect on the number of antlerless deer harvested. Our findings show that commonly applied changes in harvest regulations have a weak effect on the number of antlerless deer harvested, highlighting the challenges facing deer managers in the Midwestern US.

## Introduction

In the United States of America (US), the management of terrestrial and aquatic wildlife is generally the responsibility of each state and is guided by the principles of the North American Model of Wildlife Conservation [1]. Though laws and regulations vary among states, the implicit and explicit management goals are similar and generally fall into two broad categories: maintaining healthy wildlife populations and mitigating wildlife conflict [1]. Maintaining healthy wildlife populations can be motivated by many different factors, including the implicit value of any given species, federal mandates such as Endangered Species Act protections, recreational opportunities for the public (e.g., hunting and wildlife viewing), and economic value (e.g., tourism, equipment sales for wildlife-related recreation). Mitigating wildlife conflict mainly falls into two categories: protecting human safety and protecting the economic interests of the state and its citizens. Human safety risks that need to be mitigated include vehicle collisions [2–4], zoonotic disease transmission [2,5,6], and direct attacks [2]. Risks to economic interests include crop damage and livestock depredation [7] and disease transmission to livestock. To balance these sometimes conflicting objectives, wildlife managers need to have effective tools with predictable outcomes to respond to changes in the landscape, human and wildlife demographics, socio-political climates and diseases [8,9].

Recreational hunting is the most common and preferred tool used to manipulate white-tailed deer (*Odocoileus virginianus*) populations in the US and manage conflicts arising from high deer populations, such as habitat degradation, vehicle collisions, and crop depredation [10–14]. The main regulatory levers available to increase harvest by recreational hunters include increasing the allowed take and altering hunting season (hereafter season) length and timing [9]. These methods generally focus on the harvest of antlerless animals due to the greater impact that removing females has on population demographics [15].

An increasing challenge for wildlife managers of cervid populations, including white-tailed deer, is chronic wasting disease (CWD). CWD is a neurodegenerative disease of cervids that results from misfolded proteins called prions and is always fatal to infected individuals [16,17]. Transmission can occur directly during close contact, indirectly from prions shed into the environment where they can remain infectious for decades, and even vertically between mother and offspring [16,17]. CWD is spread more rapidly in high-density populations [16]. In addition to harming the health of individual animals, uncontrolled CWD outbreaks have the potential to cause declines in cervid populations [18,19]. The presence of CWD can also decrease interest in hunting, reducing wildlife-related recreation, economic activity that hunters generate, and license sales revenue, which is vital to funding state wildlife agencies; however, this effect appears to attenuate with time since first detection [20,21]. Minimizing the spread and prevalence of CWD is important to protect wildlife health, conservation, and economic interests of a region. Because the spread of CWD can be affected by population density, the effect of antlerless harvest regulations on deer abundance has increased importance for states that have detected CWD.

Using a large data set spanning multiple states and population management goals, we examined the effects of harvest regulations on realized antlerless deer harvest in eight Midwestern states. This information will aid managers in evaluating the effectiveness of antlerless harvest regulations, crafting future harvest regulations, and assessing if population objectives can be achieved with hunter-harvest as it is currently implemented.

## Methods

**Fig 1:**
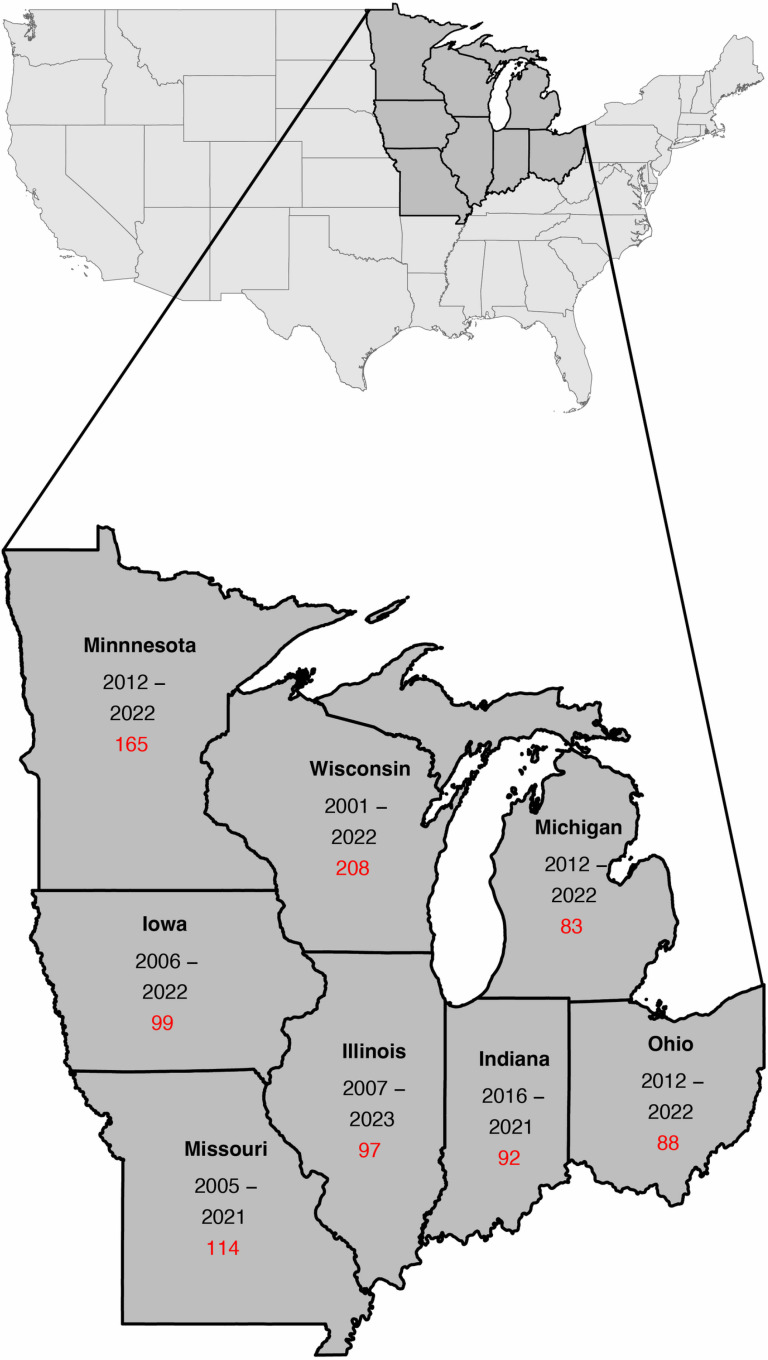
Study area. Our study area encompassed eight states in the Midwestern United States of America: Illinois, Indiana, Iowa, Michigan, Minnesota, Missouri, Ohio, and Wisconsin. Within each state, the date range of analyzed data is listed below the state name along with the number of analysis units we used. Analysis unit differed from management units as described in the methods regarding spatial change of support calculations that were needed. Additionally, in Wisconsin, data was split into two time periods: 2001–2013 and 2014–2022, with 127 analysis units in the first period and 81 in the second for the 208 listed on the map.

We collected annual deer harvest and harvest regulations data from eight Midwestern states (Illinois, Indiana, Iowa, Michigan, Minnesota, Missouri, Ohio, and Wisconsin, Fig 1). State wildlife managers provided annual harvest estimates, bag or quota limits, season lengths, and additional harvest incentive programs for each deer management unit within the state. Annual harvest data collection methodology varied from state to state but represented the best available data in all cases. Each state also provided the statewide annual number of licensed deer hunters. The harvest dataset time frames varied depending on data availability and consistency in management and reporting structure (Table 1). This study focused on antlerless deer, a definition that varies regulatorily between states but primarily encompasses females of all age classes and males under 1 year.

**Table 1:** Tag allocation systems that were used for analysis in each state with the corresponding years of antlerless harvest records. Cells marked with an asterisk (*) indicate that the relevant bag limit was invariant throughout the data set which resulted in no effect size estimate, but still allowed for an intercept estimate.

	Antlerless bag	Antlerless quota	Either sex tags available	Earn a buck	Unlimited units	First year	Last year
Illinois		x	x		x	2007	2023
Indiana	x					2016	2021
Iowa		x	x			2006	2022
Michigan	x*	x	x		x	2012	2022
Minnesota	x	x	x		x	2012	2022
Missouri	x		x*		x	2005	2021
Ohio	x*		x			2011	2022
Wisconsin		x	x	x	x	2001	2022

We evaluated how four different systems of allocating antlerless permits affected antlerless deer harvest: 1) bag limit: A specified number of permits available to each licensed hunter; 2) quota: A specified total number of permits available for a given management unit (hereafter, unit); 3) unlimited: No limit to the number of permits that could be issued to an individual hunter for use within a given unit; and 4) earn-a-buck (EAB): An incentive program wherein a hunter must harvest an antlerless deer before they can harvest an antlered deer. This system was only implemented in Wisconsin and had no limits on additional antlerless permits. In the two limited systems (bag limit and quota), we estimated the effect of changes in the number of available antlerless permits. In the two unlimited systems (unlimited and EAB), we estimated how the number of consecutive years a unit has been in the respective system affected harvest to evaluate if there was increasing or waning response with time. Across all systems, we estimated the effect of changes in available either-sex permits (permits that can be used to harvest either an antlered or antlerless deer), season lengths, and the number of licensed deer hunters. Finally, we included a regional (across all states) annual effect to control for any inter-annual variation not captured in our selected variables (Table 2). We excluded special hunt areas or units wherein hunting access and opportunity were managed outside the norms of statewide harvest management. These atypical areas either aimed to achieve a hyper-local result or limited hunter access for reasons other than manipulating harvest (e.g., military bases, multi-use state and local parks).

**Table 2:** The median value of the mean and standard deviation for the parameters across all units. Values are only listed at the configuration that they were used in the analysis. Thus, season length and the number of hunters are only reported for all units, while all of the limits for the various tag allocation systems are only reported within their respective rows.

Representative unit	Median Limit	SD Limit	Median Harvest	SD Harvest	Median Season Length	SD Season Length	Median Number of Hunters	SD Hunters
Bag Limit	1.67	0.84	699.65	113.73				
Quota	960.07	40.36	558.57	148.74				
EAB			1,708.85	582.38				
Either Sex (bag limit)	0.55	0.47	831.85	40.25				
Either Sex (quota limit)	829.41	34.68	404.59	98.14				
Full Data Set			662.53	48.34	15.69	2	47,345	7,675
Wisconsin*	3,233.33	1,617.33	1,356.96	66.91				

Across all states, the primary reason for liberalizing harvest regulations was to increase antlerless deer harvest to slow population growth or reduce deer abundance. Compared to other harvest methods (e.g., archery, muzzleloader) modern firearm harvest accounted for the most antlerless harvest in the region; therefore, we focused our analysis on antlerless harvest regulations and harvest during seasons open to firearm use. The definition of a firearm varied among states, with some states limiting hunters to shotguns or rifles using straight-walled cartridges. Additionally, the number of hunters in the dataset was restricted to those who held permits valid for firearm harvest. Antlerless harvest limits included an either-sex bag limit in seven states and an antlerless bag or quota limit in all eight states.

**Fig 2:**
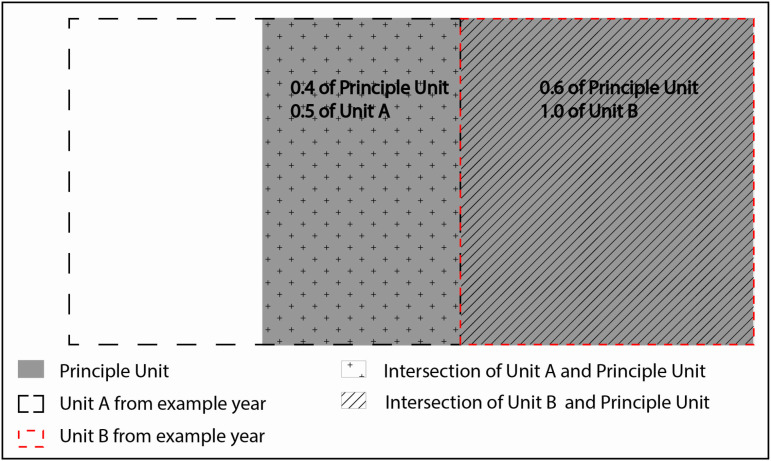
Spatial Change of support. Harvest and quota were apportioned spatially based on the portion of the original units (A and B) that overlap the new principal unit using the following equation: $Harvest\;Principle\;Unit=\sum_{i}\frac{{Area\;of\;Original\;Unit}_{i}\cap Area\;of\;Principle\;Unit}{Area\;of\;Original\;Unit}*{Harvest\;Original\;Unit}_{i}$ where i ∈{A, B}. For example, if 100 animals were harvested in each unit A and B, we assume a uniform distribution of those harvests and calculate that 150 animals were harvested in the principal unit (150=0.5*100+1.0*100). In contrast, bag limits and season lengths were apportioned based on the portion of the principal unit covered by the original unit. $Bag\;Principle\;Unit=\sum_{i}\frac{{Area\;of\;Original\;Unit}_{i}\cap Area\;of\;Principle\;Unit}{Area\;of\;Principle\;Unit}*{Bag\;Original\;Unit}_{i}$ Therefore, if the bag limit is 2 in unit A and 3 in unit B, the bag limit in the principal unit was estimated as 2.6. (2.6=0.4*2+0.6*3).

In Indiana, Iowa, Michigan, Minnesota, and Wisconsin, unit boundaries changed at least once within the study timeframe or had different harvest regulations for smaller sub-units within a larger unit. In the latter case, harvest was still reported at the unit level, so the sub-unit regulations were incorporated into the unit’s characteristics. Many of these boundary changes were part of broader strategies to respond to CWD or other management concerns that were concurrent with efforts to increase harvest, making these units important for this study. Therefore, we apportioned harvest, season lengths, and bag limit/quotas by area and calculated a change of support to use a single unit definition for the duration of each state’s data set. To do this, the unit boundaries used in the final year of each data set were defined as the principal unit boundaries. Harvest numbers, season length, and bag limit/quota values were apportioned spatially based on the overlap of the principal unit and each year’s configuration of units (Fig 2). When a principal unit combined units with different allocation systems within a year (bag, quota, unlimited, EAB), the principal unit was split into as many sub-units as needed to maintain a consistent allocation system. Wisconsin altered every unit boundary in 2014 and aligned them with county boundaries, making a change of support impractical; therefore, the spatial definitions of the data set were split into 2001–2013 and 2014–2022. Spatial change of support was carried out as described above separately for the two time frames in Wisconsin.

Antlerless harvest from each unit for each year and state was combined into a single dataset, and each harvest value was standardized at the management unit level as follows:


$$\frac{x_{[i]}-\mu_{[Unit[i]]}}{\sigma_{\left[Unit[i]\right]}}$$ (1)

where x_[i]_ is the *i*^th^ observation of harvest from the dataset, μ_[Unit[i]]_ is the mean harvest across all observed years of the unit in which the *i*^th^ observation occurs, and σ_[Unit[i]]_ is the standard deviation of observed harvest across all observed years for that unit. This allowed us to compare units that differed greatly in size and deer density, as well as factors that could differentially affect hunter success, like land access, without having to explicitly model those factors. This allowed us to detect how harvest management changes affected individual units using a common scale across all units and states. This procedure was also carried out for all continuous covariates (bag limit, quota, season length, the number of either-sex permits, and number of licensed firearm hunters). However, the number of firearm hunters was standardized at the state level, because the data were only available consistently at that resolution.

We used a Bayesian hierarchical model to examine the impact of the different management systems and other covariates on observed antlerless harvest. Specifically, we modeled


$${AL.Harvest}_{i}\sim N(\mu_{i},\;\sigma^{2})$$ (2)

where AL.Harvest_i_ is the *i*^th^ observation of antlerless harvest (in the combined dataset), which was modeled using a normal distribution with mean, μ_i_, and variance, σ^2^. Next, we modeled the mean harvest as:


$$\mu_{i}=\left(\beta_{int.B[s]}+\sum_{k}\beta_{B[s,k]}*{B\left(x_{B[i]}\right)}_{[k]}\right)I_{B[i]}+\left(\beta_{int.Q[s]}+\sum_{l}\beta_{Q[s,l]}*{B\left(x_{Q[i]}\right)}_{[l]}\right)I_{Q[i]}\;+\;\left[\beta_{int.U[s]}+\sum_{k}\beta_{T\Delta U[s,m]}*{B\left(x_{T\Delta U[i]}\right)}_{[m]}\right]I_{U[i]}+\left[\beta_{int.EAB}+\sum_{l}\beta_{T\Delta EAB[n]}*{B\left(x_{T\Delta EAB[i]}\right)}_{[n]}\right]I_{EAB[i]}+\sum_{m}\beta_{SL[s,o]}*{B\left(x_{SL[i]}\right)}_{[o]}+\sum_{n}{(\beta }_{Year[p]}*{B\left(x_{Year[i]}\right)}_{[p]}+\beta_{ES[s]}*x_{ES[i]}\;+{\;\beta }_{N.hunters[s]}*x_{N.hunters[i]},$$

where β_int.B_, β_int.Q_, β_int.U_, and β_int.EAB_ are the estimated global mean harvest values for units where permits are allocated by bag limit, quota, unlimited, and EAB, respectively. Subscript *s* identifies the state for effects estimated at the individual state level. β_B_ and β_Q_ are the estimated effects of bag and quota limit, and x_B_ and x_Q_ are the observed bag size and quota limits. The subscript *i* denotes that data and estimated harvest are for the *i*^th^ observation. Each observation is for a single unit in a single year. β_TΔU_ and β_TΔEAB_ are the estimated effects of the number of seasons a unit has been either unlimited or EAB. B(.) denotes a basis spline function with either k, l, m, n, o, or p number of knots. The x_TΔU_ and x_TΔEAB_ are the observed number of consecutive seasons a unit was either unlimited or EAB. I_B_, I_Q_, I_U_, and I_EAB_ are indicator variables controlling the inclusion of estimates associated with bag limit, quota, unlimited, and EAB units, respectively. Β_SL_ is the estimated effect of season length, and x_SL_ is the observed season length. Β_Year_ is the estimated annual effect and x_Year_ is the observed year. Β_ES_ is the estimated effect of either-sex permits, and x_ES_ is the observed available either-sex permits. β_N.hunters_ is the estimated effect of the number of licensed firearm hunters, and x_N.hunters_ is the observed number of licensed firearm hunters.

We hypothesized that season length, bag limits, and quota limits would have a non-linear relationship with annual harvest. Therefore, we used a 1-degree B-spline function to model the standardized data with a sum-to-zero constraint to permit estimation of the intercept terms [22]. For season length, we used eight internal knots to describe the potential non-linearity and were placed at quantiles 0.03, 0.1, 0.2, 0.4, 0.6, 0.8, 0.9, and 0.97. Bag and quota limits were described using three internal knots at quantiles 0.15, 0.5, and 0.85. The annual effect and the two time-varying effects (seasons as EAB, seasons as unlimited) were also modeled using 1-degree B-splines, with a knot for each year between 1 and the maximum number of years observed to allow for a continuous non-linear estimate of the effect of time.

For the intercept terms describing the mean harvest associated with each allocation system in Equation 2, we specified a diffuse, normal prior distribution with a mean of zero and variance, v_int_. We specified an exponential prior distribution for v_int_ with a rate equal to the square root of 5. Shrinkage priors were used for all remaining parameters [23]. We selected a zero mean Laplace distribution shrinkage prior with an inverse gamma (0.1, 0.1) prior distribution for the associated variance. Similar priors were used as appropriate for parameters for the B-spline basis functions.

Given the standardization of the harvest response described above, the estimated parameters detail the effects of the standardized covariates on the standard deviation scale of harvest. Therefore, to create more easily interpreted estimates of the effects of covariates on harvest, we determined the median value of mean (μ) and standard deviation values (σ) used in Equation 1 across all deer management units within each of the tag allocation systems and for season length and number of hunters. We then used these median values and the parameter posterior distributions to calculate the impact of the changes in levels of tag allocations, season length and number of hunters on harvest (Table 2).

To obtain regional estimates of the effects of the different systems and other covariates, we averaged the estimated state-specific parameters during each MCMC iteration. We present regional results for the effects of bag and quota limits by only averaging effects where all states had data.

We obtained posterior distributions of our coefficients using the nimble package implemented in R [24–26]. We ran three MCMC chains for 200,000 iterations, with 20,000 iterations removed as burn-in. Evidence for non-convergence was assessed using trace plots and the Gelman-Rubin diagnostic [27]. Model fit was evaluated using a posterior predictive check described in Kéry & Royle, 2016 Kéry & Royle, 2016 [28]. An expected, antlerless harvest was drawn from a normal distribution for each observation i, with mean, μ_i_, and variance, σ^2^ both of which were estimated in the model. Two chi-squared discrepancies were then calculated comparing μ_i_ to the observed antlerless harvest (AL.Harvest_i_) and the expected antlerless harvest. These values were then summed within each MCMC iteration and plotted against each other for a visual check of fit along a 1–1 line. Points falling near the 1–1 line indicate good fit. We calculated a Bayesian p-value as the probability that the chi-squared value of the expected antlerless harvest was greater than the chi-squared value of the observed antlerless harvest.

## Results

### Model convergence and fit

All chains and parameters showed no evidence of non-convergence both graphically and with Gelman-Rubin diagnostic values of less than 1.01. Model fit testing gave a Bayesian P-value of 0.55 and plotted $\chi^{2}$ values generally fell along the 1–1 line (S1 Fig).

### Limited allocation systems

**Fig 3:**
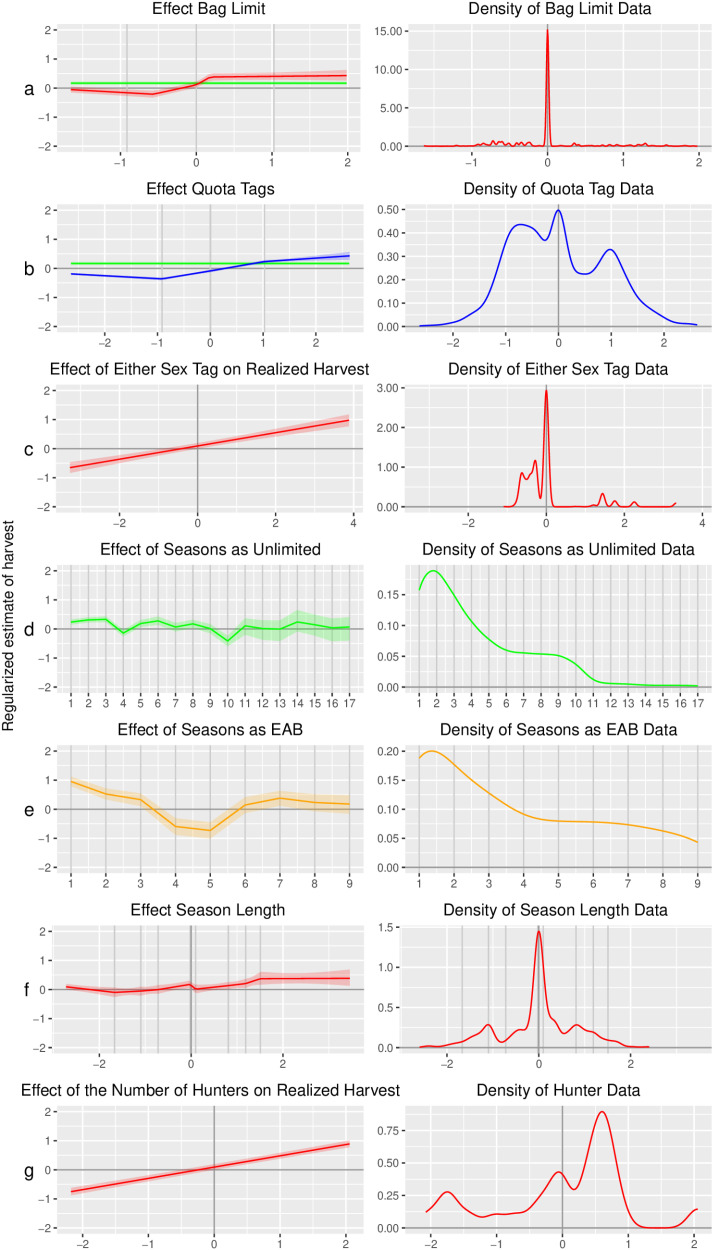
Model estimates and data density. The left column shows model-estimated regional effects of the seven management covariates we considered in this paper. The y-axis of all seven graphs in the left column is standardized by subtracting the mean harvest and dividing by the standard deviation of the harvest for each management unit. The effect of each covariate is shown with all other covariates held at their average value. Red lines represent estimates assuming a bag limit permit allocation system in rows a, c, f, and g and slope in panel a. The horizontal green line in panels a and b is the effect of unlimited permit units to illustrate the point at which increasing bag limits or permit availability creates the same effect as an unlimited allocation. The x-axis in rows a, b, c, f, and g are based on standardized covariate values. In rows d and e, the x-axes are discrete years starting with the first year a unit was either unlimited or EAB. The right column shows the data density for each covariate. The plots in rows a and b have been clipped to only the range where data overlapped for all states to better present a regional perspective, estimates were still derived using all available data. Key for permit allocation system: Red = Bag limited, Blue = Quota limited, Green = Unlimited, Gold = Earn-a-buck..

**Fig 4:**
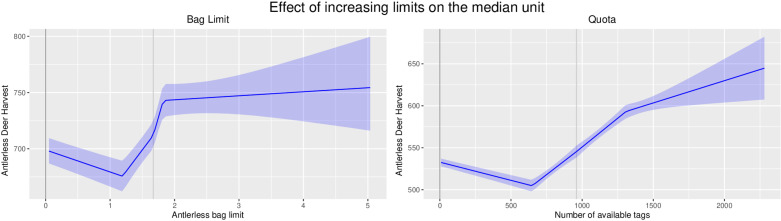
Effect of changing harvest limits on a median unit.The estimated antlerless deer harvest for different bag limits or quotas respectively when the model is applied to the median unit from the data set for both tag allocation systems. The grey vertical line in both plots is the average bag limit or quota for the median unit described in this figure. This line corresponds to zero on the x-axis in the plots in Fig 3, where the results are reported as standard deviations from a unit’s mean.

Increasing the number of available permits or the bag size for the quota and bag limit systems, respectively, resulted in a small increase in harvest in each state and associated small increases in the regional estimates of harvest (Figs 3, S2 and S3). All reported estimates are based on the mean value of the posterior distribution. Increasing a bag limit by 1 standard deviation from a unit’s average bag limit resulted in an estimated 0.29 (95% CI 0.19–0.40) standard deviation increase in that unit’s antlerless harvest. In the representative bag limit unit, this corresponded to an increase of the antlerless bag limit from 1.67 to 2.51 deer, resulting in an increase in the harvest of 33 animals (95% CI 22–46, Table 2, Fig 4) from 700 to 733. This is a 5% (95% CI 3%-7%) increase in harvest for a 50% increase in the bag limit. Harvest showed a substantially lower response to an increase in bag limits as limits increased further away from the unit mean (Figs 3 and 4). Similarly, increasing a quota by 1 standard deviation from a unit’s average quota resulted in an estimated 0.24 (95% CI 0.16–0.31) standard deviation increase in that unit’s antlerless harvest. This corresponds to adding 340 permits to the average 960 permits in the representative quota unit, which would result in an additional 35 animals (95% CI 27–42) being harvested above the average harvest of 559 antlerless deer (Table 2, Fig 4). This is a 6% (95% CI 5%-8%) increase in harvest for a 35% increase in quota per unit. Both bag limit and quota showed important non-linear features, and most notably, harvest was most responsive to changes in the bag limit or quota when the changes occurred close to the unit’s average limit (Fig 3). For example, all increases in bag limit above 0.18 standard deviations from the unit’s mean bag limit do not result in statistically different increases in harvest (Fig 3). In the representative unit, this means that an increase from a bag limit of 1.67 to 1.82 is not discernable from an increase to a bag limit of 2, 3, or 4. In deer management units using quota, the effect of increasing quota continues to have a positive effect beyond an increase of 1.02 standard deviations, but the rate of increase declines, and the variability in the harvest response increases substantially (Fig 3).

The lowest observed bag limits and quotas showed an interesting, slightly negative relationship with harvest, wherein harvest increased with decreasing limits. The negative effect on the lowest limits in bag limit units is small with an effect size of -0.145 (95% CI -0.156- -0.136), which would result in an increase in harvest of 16 deer with 1 standard deviation decrease in the bag limit, which is 2% of the harvest for the representative bag unit. The negative effect on the lowest limits in quota units is minimal with an effect size of -0.041 (95% CI -0.0426 - -0.040), which would result in an increase in harvest of 5 deer with 1 standard deviation decrease in quota, which is less than 0.5% of the harvest for the representative quota unit.

Either-sex permit allocations also had a positive relationship with realized antlerless harvest (Figs 3 and S4). An increase in 1 standard deviation of the number of either-sex permits available in a unit resulted in an estimated 0.23 standard deviation (95% CI 0.18–0.27) increase in antlerless harvest. We observed both bag limits and quotas for administering either-sex permits. Therefore, an increase of an either-sex bag limit from 0.55 to 1.01 would increase the estimated average harvest of 832 antlerless deer by 55 deer (95% CI 43–66, Table 2) in our representative unit. This is a 7% (95% CI 5%-8%) increase in harvest for an 86% increase in the either-sex bag limit per unit. If the either-sex bag limit was raised by 1 (2.13 SD), an increase in bag limit of 183%, harvest would increase by 117 animals (95% CI 92–141), a 14% increase in harvest. An increase of an either-sex quota from 829 to 1,064 would increase the estimated average harvest of 405 antlerless deer by 22 deer (95% CI 18–27, Table 2) in our representative unit. This is a 6% (95% CI 4%-7%) increase in harvest for a 28% increase in the either-sex quota per unit.

### Unlimited and EAB systems

Unlimited harvest permit allocation resulted in a higher harvest than the average for both bag limit and quota allocations. However, the posterior distributions for intercept estimates for both bag limit and unlimited permit allocations overlap significantly. Interestingly, by raising the bag limit approximately 0.06 standard deviations above the average bag limit, the resulting estimated unit-level harvest is equal to the estimated harvest under an unlimited allocation (Figs 3 and S2). This suggests that raising the bag limit of the representative unit by 0.05 deer would result in the same or greater harvest than using an unlimited harvest allocation; however, it is worth noting that due to the bag limit structure, the minimal increase in the bag limit is 1, which indicates that any increase in the bag limit from the units average limit will exceed the effect of unlimited allocation. This same equality occurs at approximately 0.8 standard deviations above average for quota permit allocations (272 additional antlerless permits for the representative quota unit; Figs 3 and S3).

EAB only occurred in Wisconsin, where it resulted in a substantially higher harvest than either unlimited or quota-limited permit allocation. When EAB is in effect, harvest in the representative EAB unit is estimated to increase by 211 more antlerless deer than the unit average of 1,709 (95% CI 149–261), for a 12% increase in harvest. Our model found that a quota would have to be raised 1.9 standard deviations from its average quota to realize the same harvest increase as EAB (S3 Fig). This would mean raising the quota by 3,072 additional antlerless permits from the average in the Wisconsin representative unit, a 95% increase in quota (Table 2).

The number of seasons that a unit was managed under an unlimited permit allocation system had a negligible effect on antlerless harvest (Figs 3 and S5). However, EAB units started above the average estimated EAB harvest, but steadily decreased through year 3 with harvest dropping below average for years 4 and 5, before returning to above average for years 6–9 (Fig 3). The estimated harvest in year 5 of EAB drops below the estimated average harvest in units using an average quota permit allocation in Wisconsin.

### Season length

Changes in season length had a nonlinear relationship with realized harvest. The general trend across the states was positive; however, estimates varied widely among states suggesting context-dependent relationships in adding additional seasons or lengthening existing ones (Figs 3 and S6 Fig).

### Hunter numbers

As expected, increasing hunter numbers increased the realized harvest (Figs 3 and S7). This occurred at differing rates among states without a notable trend associated with the permit allocation system (bag limit, quota, unlimited, or EAB). A 1 standard deviation increase in the number of firearm hunters in a state resulted in a 0.39 (95% CI 0.35–0.42) standard deviation increase in antlerless harvest within a unit. Median hunter participation across all eight states was 347,345 firearm hunters, with a median standard deviation of 17,675 hunters (Table 2). Therefore, with the addition of 17,675 hunters statewide (5% increase in hunters), the representative unit harvest would increase by 58 (95% CI 52–63) antlerless deer from 663 to 720, or an increase in harvest of 9% (95% CI 8%-10%). With a median of 98 units per state, this could equate to statewide changes in harvest of over 5,500 antlerless deer.

## Discussion

As expected, the number of antlerless harvest permits has a positive relationship with harvest. However, the effect is generally weak. Due to the nonlinear nature of the effect of altering bag and quota limits on antlerless harvest, changes in bag and quota limits had the strongest effect when changes occurred close to a unit’s average limit (between -0.57 and 0.18 SD for bag limit, and -0.91 and 1.03 SD for quota). As limits were altered beyond 0.18 standard deviations for bag limits and 1.03 for quotas, the effect of changes to limits on harvest substantially diminished. This diminishing return most likely occurs because of the limited per-hunter harvest demand, which has been reported as being below two deer on average (antlered and antlerless combined) per hunter per year [29].

There was a negative relationship between harvest and increasing bag limits and quotas for the lowest observed values (0.57 standard deviations below the average for bag units and 0.91 for quota units). This effect is minor and would only result in a 0.5–2% change in harvest for either representative unit. Further research is needed to understand the underlying mechanism giving rise to this negative relationship between available permits and antlerless harvest when the limit is well below the units’ average.

Increasing unlimited permit availability had a positive effect on a unit’s antlerless harvest, but the same increase in harvest was achievable by increasing bag limits or quotas. An equivalent harvest could be achieved by increasing the median bag limit by only 0.05 per unit. A similar result is seen for quota units, where a 28% increase in the quota for the median quota unit would result in the same increase in antlerless harvest as issuing unlimited permits. Some states saw greater increases in a unit’s antlerless harvest when changing to an unlimited allocation system, but all states saw a negligible change in this effect on harvest over time. Therefore, raising limited permit allocations may be more effective at increasing antlerless harvest than unlimited permit allocations and allows future increases in permits if needed.

The one incentive program we measured, EAB, showed a substantial increase in antlerless harvest that declined in the second year through the fifth year when it was below the model average for limited harvest. Harvest subsequently rebounded after year six to above the estimated average for limited harvest. This temporal variation in harvest could be attributed to variation in the size of the deer population influencing hunter success [30]; however, further analysis incorporating deer population estimates and hunter effort could clarify this relationship. EAB also experienced strong resistance from some hunters, which may be important to consider before implementation [31].

Season length had a modest impact on antlerless harvest without a notable relationship with the permit allocation system, which aligns with existing research [32,33]. It is worth noting that states differed in both the magnitude and the non-linear response of harvest to changes in season length (S6 Fig). This suggests the effects of adding or lengthening seasons were state-dependent. Our analysis could not account for the timing of additional seasons in relation to existing seasons, and environmental, or social factors. A state-level analysis of additional seasons, timing, regulations, and hunter values or attitudes could help inform future changes in harvest regulations.

As expected, hunter participation substantially impacted the realized harvest, resulting in the largest effect size of all the continuous variables we modeled. Therefore, any effort to maintain or increase hunter harvest is highly dependent on maintaining or increasing hunter numbers, which can be challenging to achieve [34,35]. The number of hunters has been trending down across all the states included in this analysis (S8 Fig). If this continues, any gains in harvest from manipulating the harvest systems employed, season structure, increasing permit availability, or altering season lengths may be eclipsed by the effect of declining hunter numbers.

Every management action we measured positively affected antlerless deer harvest. Harvest was responsive to increasing bag limits and quotas, however, the effect decreased substantially as increases in limits moved away from the unit average (Fig 3). Increases in bag limit beyond a bag of 2 are modeled to have no additional effect on harvest in a representative bag unit (Fig 4). Similarly, increases in quotas greater than 35% above the unit average will have a diminishing additional impact on the median quota unit (Fig 4). Additionally, both bag limit and quota increases above the unit’s average of less than 1 or 35%, respectively, resulted in a greater estimated antlerless harvest than unlimited permit allocation. At the conclusion of this study, 41% of bag units had bag limits at or above 0.18 standard deviations from the unit average making further increases unlikely to have any effect on harvest. Quota units had a more modest 13% of units with quotas at or above their inflection point of 1.03 standard deviations. Therefore, we believe that small changes in harvest can be achieved when making adjustments in permit allocations close to the unit’s average permit limits, but large increases in harvest are unlikely to be achieved. This is further confounded by the fact that many units’ permit limits have already reached or exceeded the level where additional increases are likely to influence antlerless harvest. These patterns also hold for decreasing harvest. Minor decreases in hunter harvest are possible, but primarily when adjustments are made close to the unit’s average.

Increasing antlerless deer harvest is predicated on a change in voluntary actions from current or prospective deer hunters. The factors that influence the individual hunter to change the number of deer they harvest or inspire someone to start hunting are beyond the scope of this paper and likely vary between and even possibly within states. However, some common factors that can influence hunter behavior as it pertains to harvest regulations include alignment of regulation changes with local hunting traditions, trust in the state wildlife agency, perception of whether herd reduction will achieve the desired outcome, and per-hunter harvest demand [29,36–39]. The factors that influence agency trust among hunters can be difficult to predict. For example, Vaske and Miller 2021 found that less than 4% of the variation in individual trust in state wildlife agencies could be predicted by demographic and individual hunting-related variables [40]. Therefore, tailoring future actions either to mobilize individuals who have high trust or to increase trust in others may be difficult. While popular as a concept, programs that facilitate hunter-harvest donations to increase per-hunter harvest present logistical difficulties (food safety testing, etc.) in CWD-positive areas, require increased demands on hunters’ time, and have seen a decline in participation in some areas [41]. While the specific human dimension factors influencing the effect of changes in harvest regulations likely vary by state, our findings were consistent: small-magnitude changes in harvest are achievable when pursued close to a unit’s median harvest level while larger magnitude changes or small changes to a unit already well outside its historical level will be difficult to achieve using traditional approaches to harvest management.

For larger magnitude increases in antlerless harvest such as desired in response to CWD, an incentive program like EAB or novel approaches may be necessary. EAB was the most effective method observed in this study for increasing hunter harvest, raising harvest by an estimated 12% per unit; however, it presents socio-political challenges with many hunters being resistant to it in principle and application [31]. Additionally, if the number of deer hunters continues to decline, even the efficacy of EAB will be negatively impacted. Finally, our model suggests there is a level of antlerless harvest that cannot be achieved with recreational hunting alone (greater than 2 standard deviations of the unit’s average harvest) as it is currently implemented. If harvest greater than this is desired, the use of novel approaches to harvest management may need to be developed or non-harvest methods such as sharpshooters or commercial harvest may be required, both of the latter may encounter resistance as they can be perceived as counter to the North American Model of Wildlife Conservation and may be unpopular with hunters and the general public. [12,42,43].

## Supporting information

S1 Fig Model fit estimating antlerless deer harvest based on harvest regulations. Each point is the summation of Chi Squared values. The X values are Chi Squared values of the model against the observed data and the Y values are the Chi Squared values of the model against an expected distribution of observations. For clarity this plot is zoomed in. The values excluded from this plot account for less than 0.1% of the posterior estimates of fit and are similarly distributed above and below the 1:1 line.
(TIF)

S2 Fig The effect of increasing antlerless deer bag limit on realized antlerless deer harvest by state. The green line is the intercept value for unlimited antlerless permit allocation when pursued in a given state. The right column shows the density distribution of data for each state. Key: Red = Bag limited, Green = Unlimited. All estimate lines are truncated to the covariate range observed for the respective permit allocation system. The data density and line truncation are provided for context and were not explicitly part of the model.
(TIF)

S3 Fig The effect of increasing the antlerless deer quota on realized antlerless deer harvest by state. The green line is the intercept value for unlimited antlerless permit allocation when pursued in a given state, and the gold line in the Wisconsin graph is the intercept value for earn-a-buck. The right column shows the density distribution of data for each state. Key: Blue = Quota limited, Green = Unlimited, Gold = Earn-a-buck. All estimate lines are truncated to the covariate range observed for the respective permit allocation system. The data density and line truncation are provided for context and were not explicitly part of the model.
(TIF)

S4 Fig The effect of increasing either-sex deer permit allocations on realized antlerless deer harvest by state. The right column shows the density distribution of data for each state. All estimate lines are truncated to the covariate range observed for the respective permit allocation system. Key: Red = Bag limited, Blue = Quota limited, Green = Unlimited, Gold = Earn-a-buck. All estimate lines are truncated to the covariate range observed for the respective permit allocation system. The data density and line truncation are provided for context and were not explicitly part of the model.
(TIF)

S5 Fig The effect of unlimited antlerless permit availability on realized antlerless deer harvest by state through time. This effect was estimated at the regional level and therefore the shape and magnitude of the line are identical among all 5 states. The unlimited intercept value for each state differs among the plots. The dips in years 4 and 10 correlate with observed reductions in harvest in Missouri due to outbreaks of hemorrhagic disease. Most of Missouri’s unlimited units were designated in the same year, and by year 4, Missouri accounted for greater than 45% of observed unlimited units and greater than 80% in year 10. We believe that this creates the two dips and rebounds from what would otherwise be a nearly flat line. The right column shows the density distribution of data for each state. All estimate lines are truncated to the covariate range observed for the respective permit allocation system. Key: Red = Bag limited, Blue = Quota limited, Green = Unlimited, Gold = Earn-a-buck. All estimate lines are truncated to the covariate range observed for the respective permit allocation system. The data density and line truncation are provided for context and were not explicitly part of the model.
(TIF)

S6 Fig The effect of increasing season length on realized antlerless deer harvest by state. The right column shows the density distribution of data for each state. All estimate lines are truncated to the covariate range observed for the respective permit allocation system. Key: Red = Bag limited, Blue = Quota limited, Green = Unlimited, Gold = Earn-a-buck. All estimate lines are truncated to the covariate range observed for the respective permit allocation system. The data density and line truncation are provided for context and were not explicitly part of the model.
(TIF)

S7 Fig The effect of changes in hunter numbers on realized antlerless deer harvest by state. The right column shows the density distribution of data for each state. All estimate lines are truncated to the covariate range observed for the respective permit allocation system. Key: Red = Bag limited, Blue = Quota limited, Green = Unlimited, Gold = Earn-a-buck. All estimate lines are truncated to the covariate range observed for the respective permit allocation system. The data density and line truncation are provided for context and were not explicitly part of the model.
(TIF)

S8 Fig Annual count of firearm deer hunters by state. (TIF)
