# Supplementary figures and images for: Doe diligence: A regional analysis of antlerless deer harvest regulations in the Midwestern United States of America
Source: PLoS One. 2025 Jun 4;20(6):e0324708. doi: 10.1371/journal.pone.0324708 (PMC12136447; doi:10.1371/journal.pone.0324708)

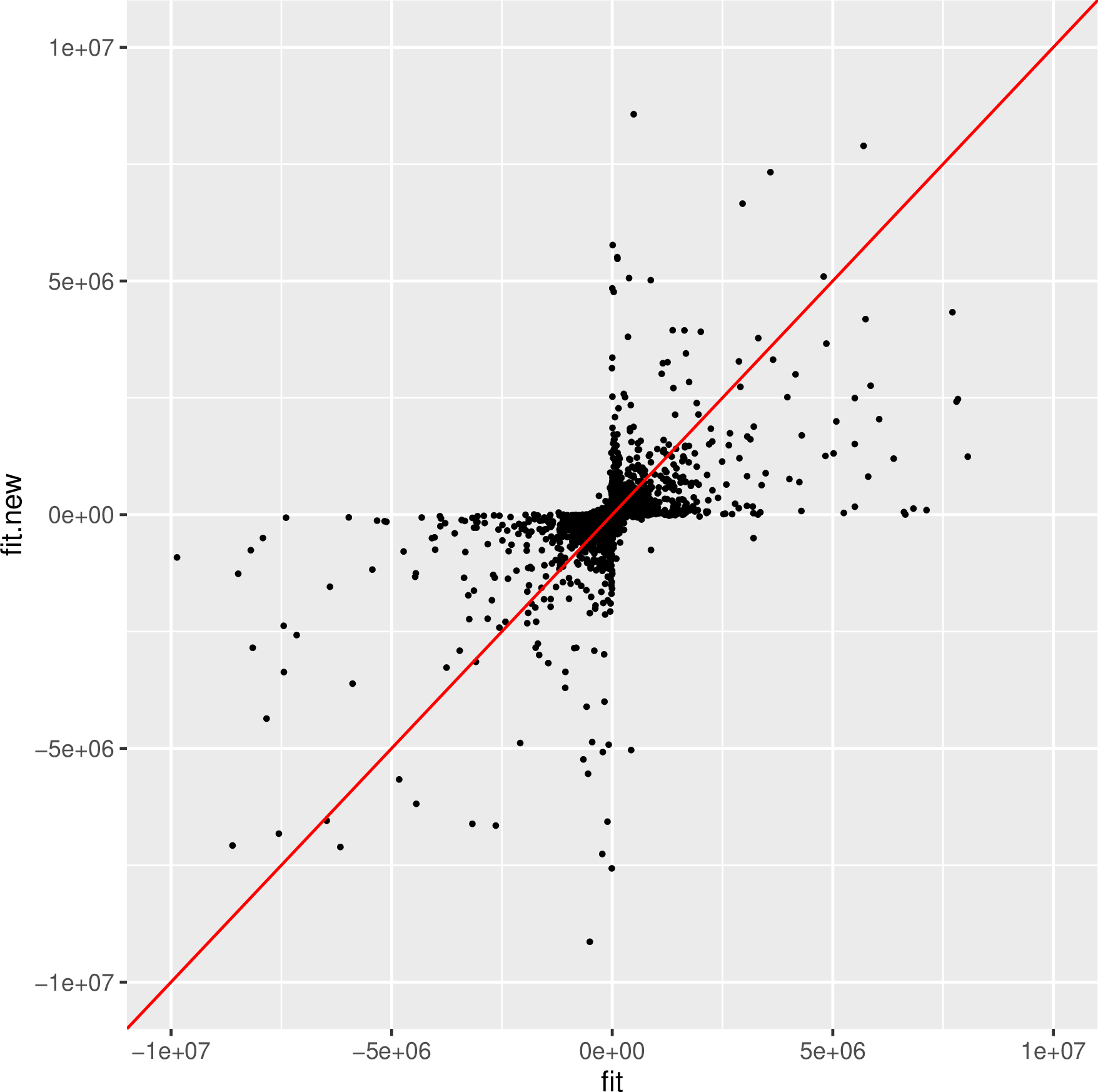

Supplement: S1 Fig — Each point is the summation of Chi Squared values. The X values are Chi Squared values of the model against the observed data and the Y values are the Chi Squared values of the model against an expected distribution of observations. For clarity this plot is zoomed in. The values excluded from this plot account for less than 0.1% of the posterior estimates of fit and are similarly distributed above and below the 1:1 line. (TIF) [file pone.0324708.s001.tif]

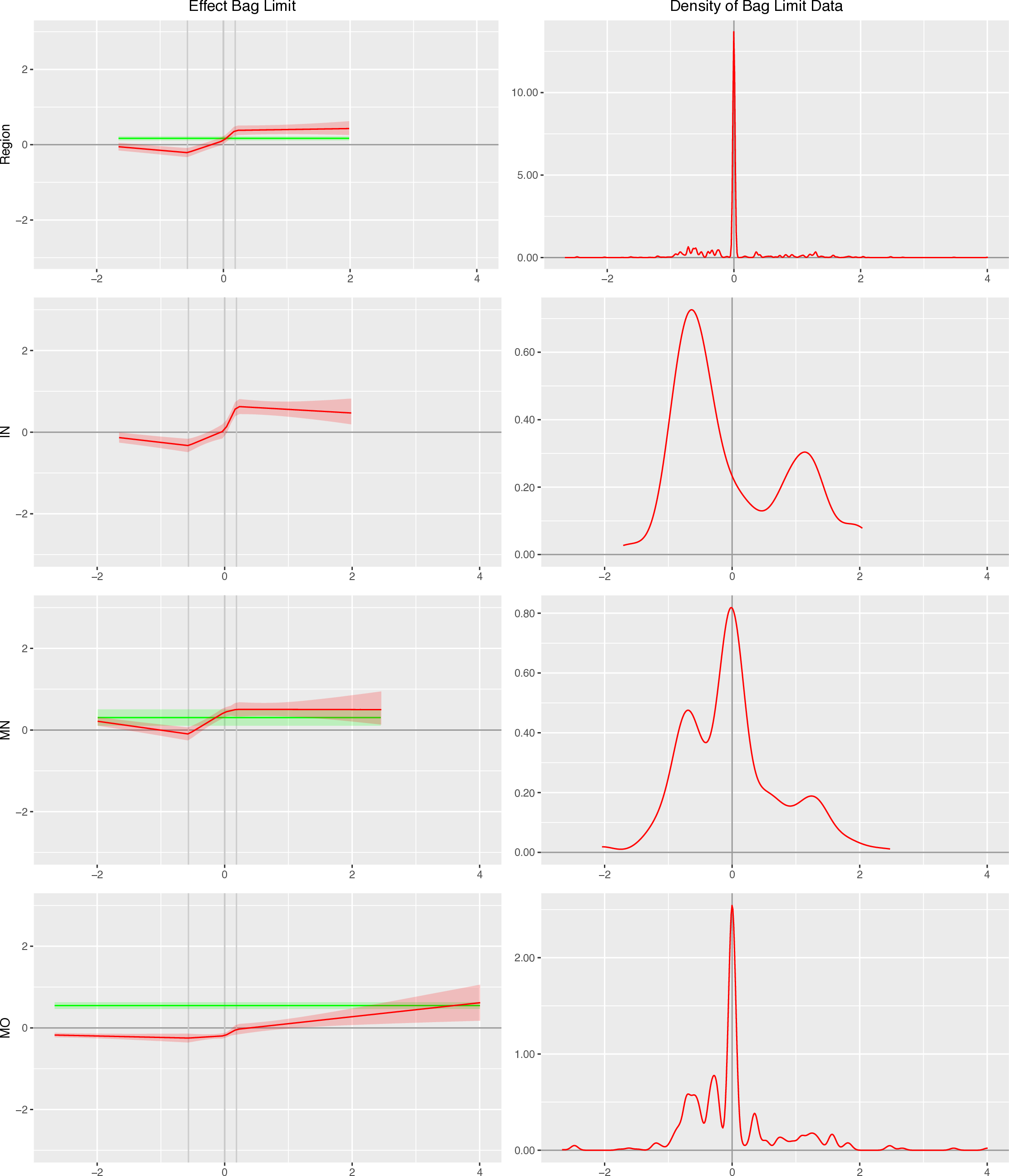

Supplement: S2 Fig — The green line is the intercept value for unlimited antlerless permit allocation when pursued in a given state. The right column shows the density distribution of data for each state. Key: Red = Bag limited, Green = Unlimited. All estimate lines are truncated to the covariate range observed for the respective permit allocation system. The data density and line truncation are provided for context and were not explicitly part of the model. (TIF) [file pone.0324708.s002.tif]

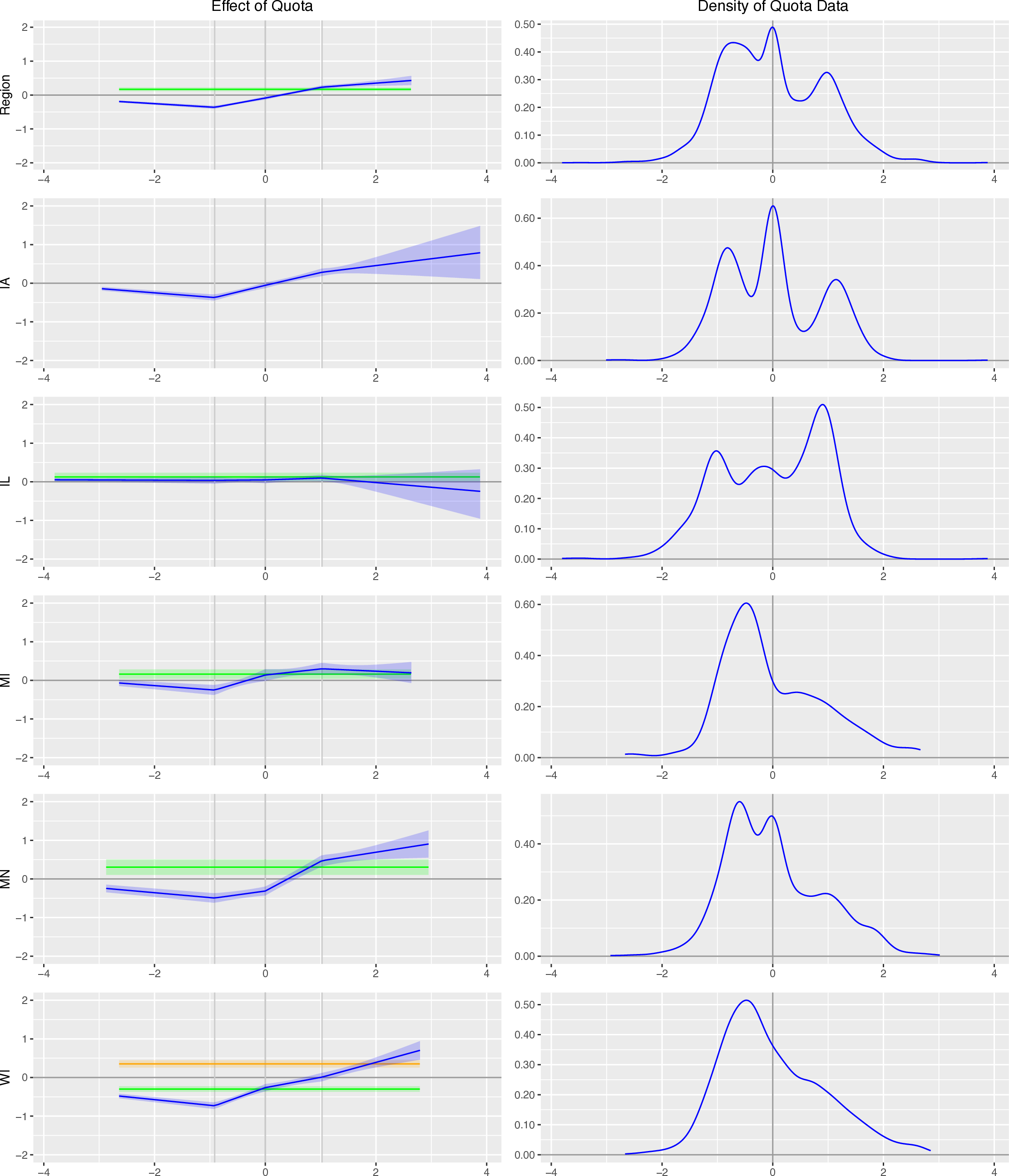

Supplement: S3 Fig — The green line is the intercept value for unlimited antlerless permit allocation when pursued in a given state, and the gold line in the Wisconsin graph is the intercept value for earn-a-buck. The right column shows the density distribution of data for each state. Key: Blue = Quota limited, Green = Unlimited, Gold = Earn-a-buck. All estimate lines are truncated to the covariate range observed for the respective permit allocation system. The data density and line truncation are provided for context and were not explicitly part of the model. (TIF) [file pone.0324708.s003.tif]

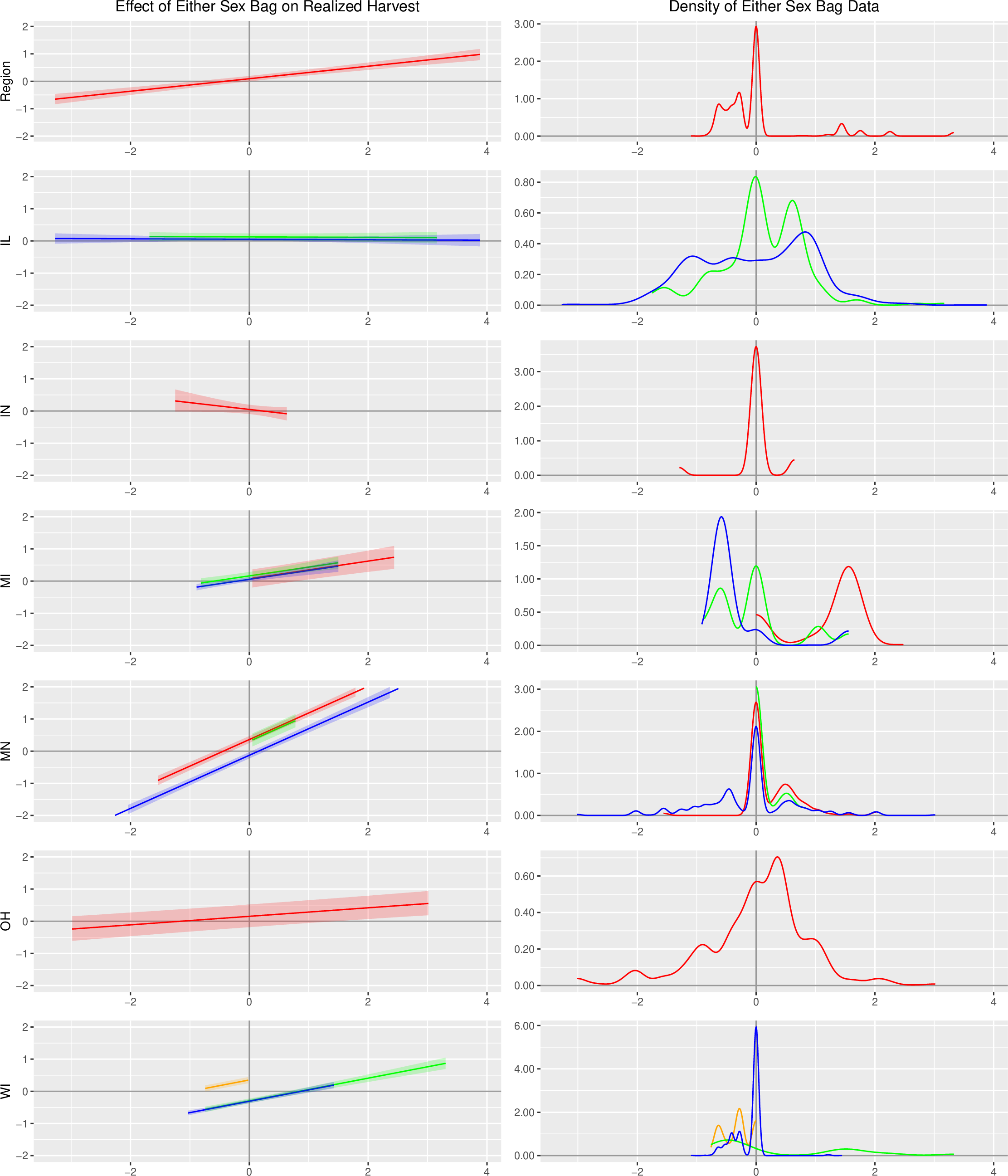

Supplement: S4 Fig — The right column shows the density distribution of data for each state. All estimate lines are truncated to the covariate range observed for the respective permit allocation system. Key: Red = Bag limited, Blue = Quota limited, Green = Unlimited, Gold = Earn-a-buck. All estimate lines are truncated to the covariate range observed for the respective permit allocation system. The data density and line truncation are provided for context and were not explicitly part of the model. (TIF) [file pone.0324708.s004.tif]

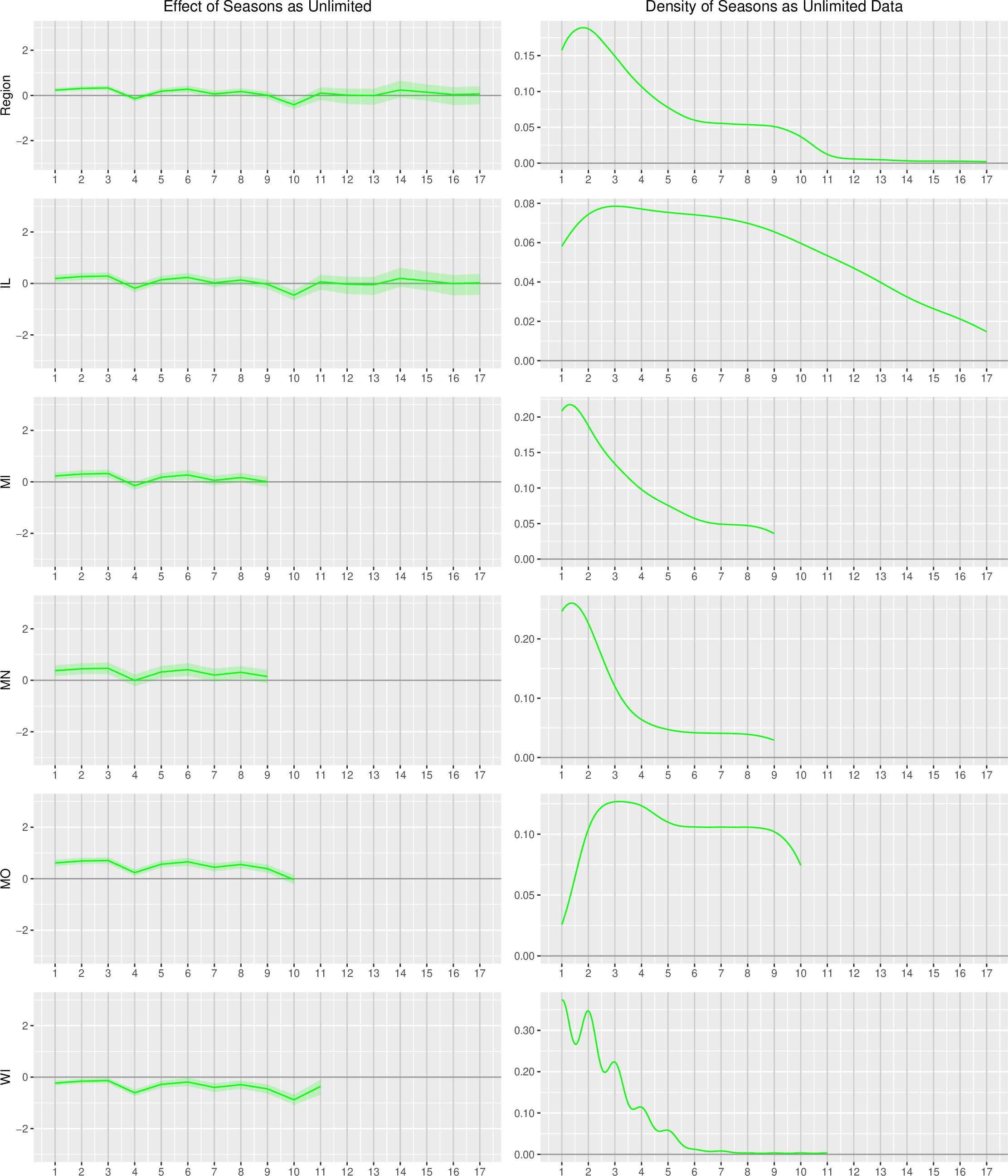

Supplement: S5 Fig — This effect was estimated at the regional level and therefore the shape and magnitude of the line are identical among all 5 states. The unlimited intercept value for each state differs among the plots. The dips in years 4 and 10 correlate with observed reductions in harvest in Missouri due to outbreaks of hemorrhagic disease. Most of Missouri’s unlimited units were designated in the same year, and by year 4, Missouri accounted for greater than 45% of observed unlimited units and greater than 80% in year 10. We believe that this creates the two dips and rebounds from what would otherwise be a nearly flat line. The right column shows the density distribution of data for each state. All estimate lines are truncated to the covariate range observed for the respective permit allocation system. Key: Red = Bag limited, Blue = Quota limited, Green = Unlimited, Gold = Earn-a-buck. All estimate lines are truncated to the covariate range observed for the respective permit allocation system. The data density and line truncation are provided for context and were not explicitly part of the model. (TIF) [file pone.0324708.s005.tif]

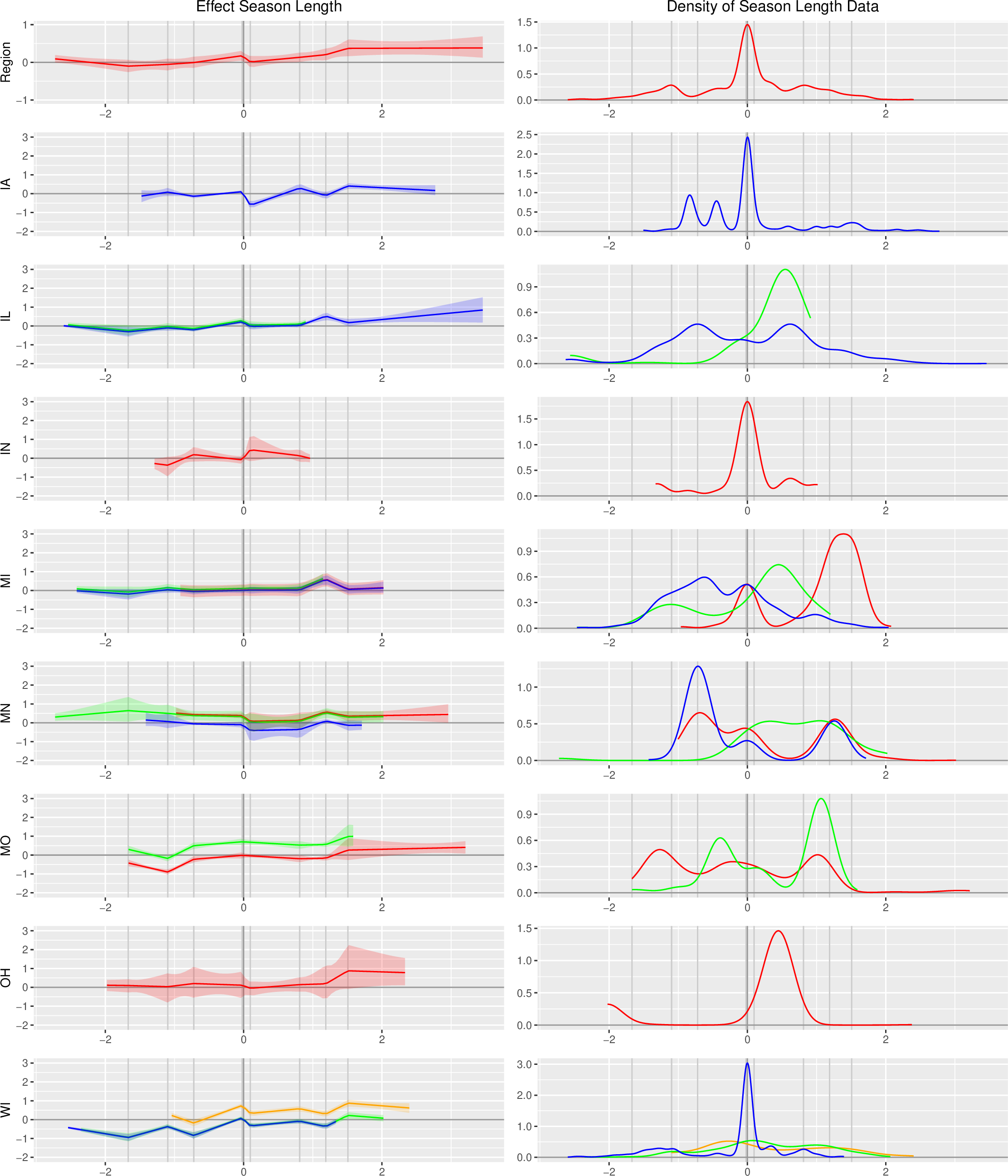

Supplement: S6 Fig — The right column shows the density distribution of data for each state. All estimate lines are truncated to the covariate range observed for the respective permit allocation system. Key: Red = Bag limited, Blue = Quota limited, Green = Unlimited, Gold = Earn-a-buck. All estimate lines are truncated to the covariate range observed for the respective permit allocation system. The data density and line truncation are provided for context and were not explicitly part of the model. (TIF) [file pone.0324708.s006.tif]

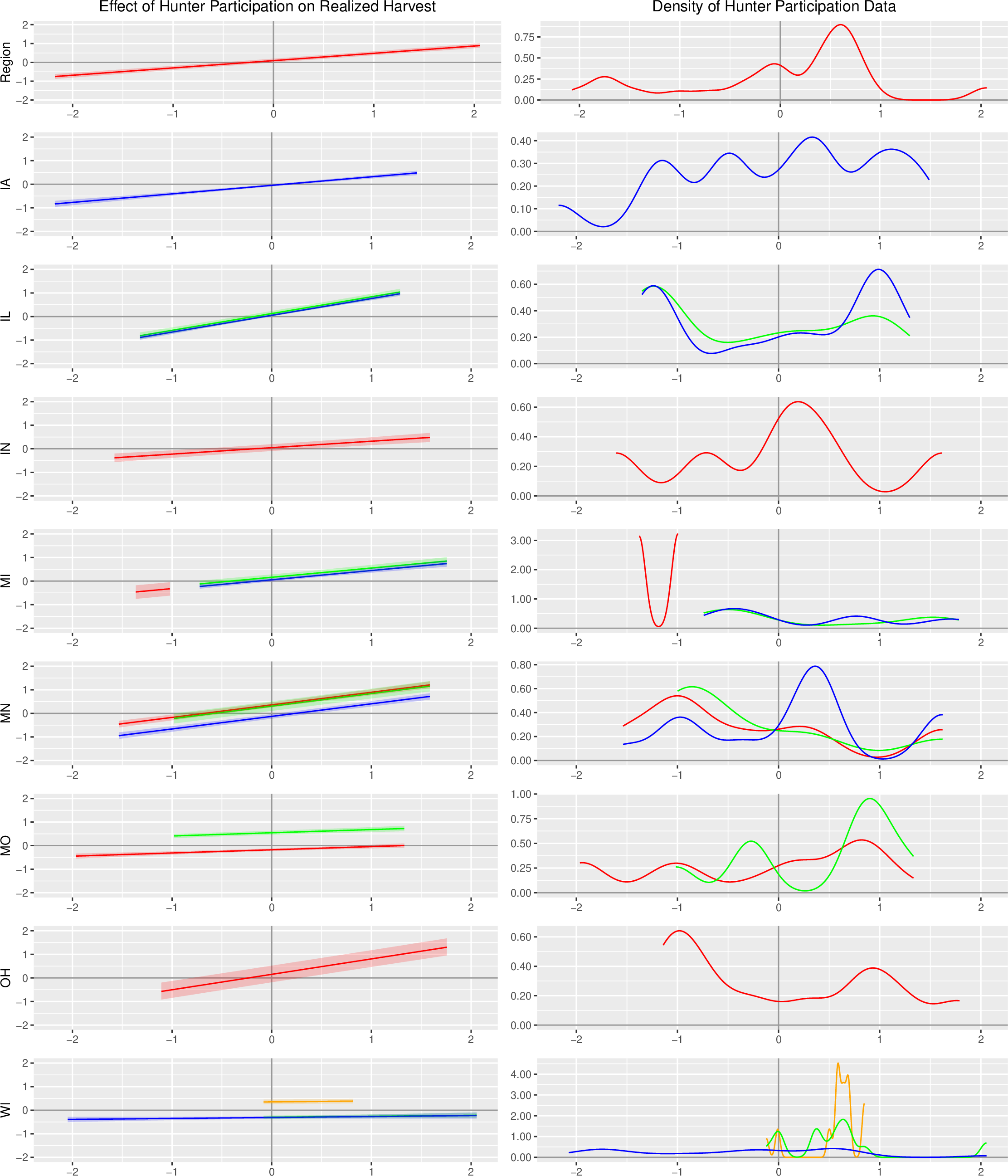

Supplement: S7 Fig — The right column shows the density distribution of data for each state. All estimate lines are truncated to the covariate range observed for the respective permit allocation system. Key: Red = Bag limited, Blue = Quota limited, Green = Unlimited, Gold = Earn-a-buck. All estimate lines are truncated to the covariate range observed for the respective permit allocation system. The data density and line truncation are provided for context and were not explicitly part of the model. (TIF) [file pone.0324708.s007.tif]

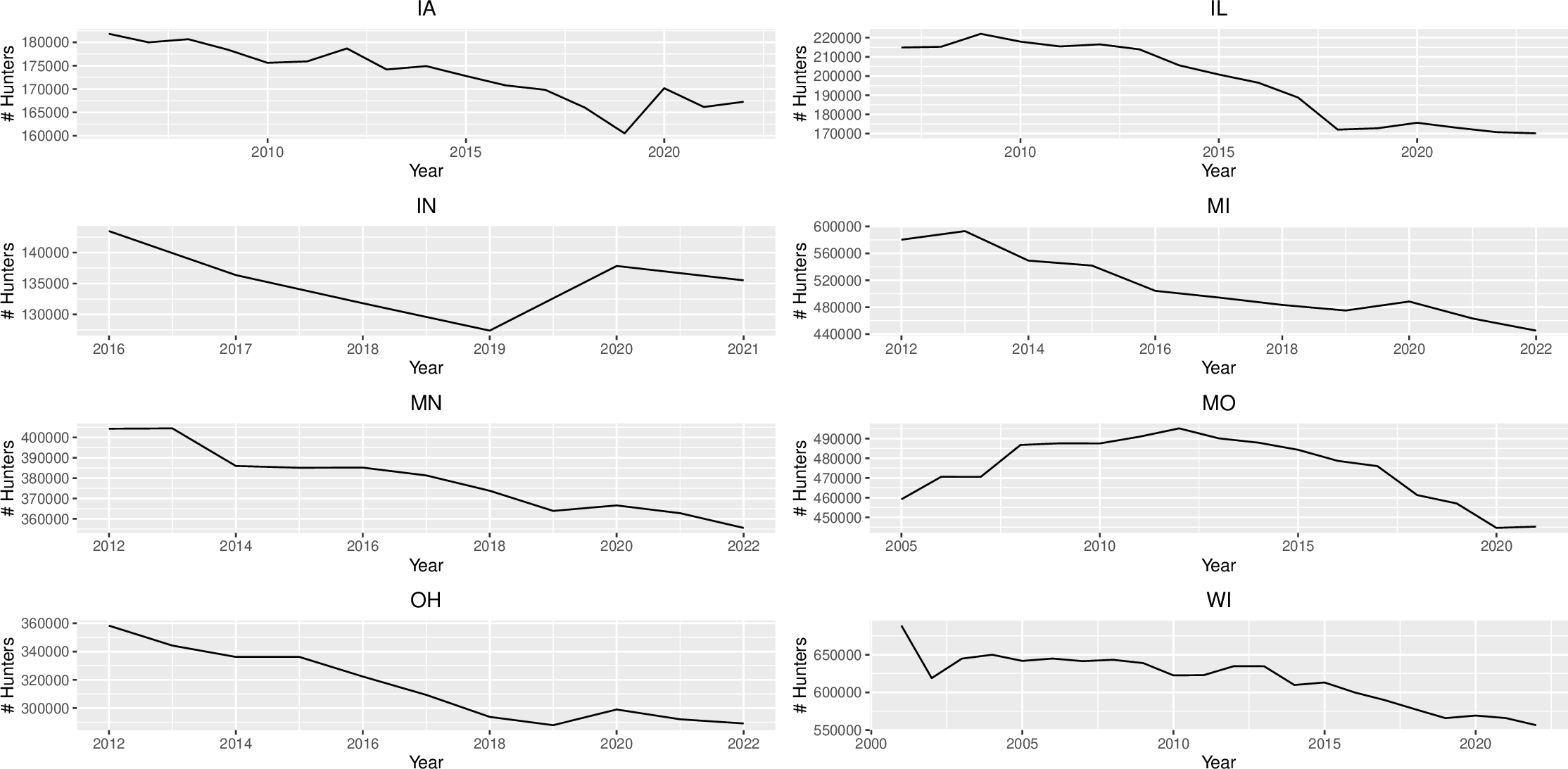

Supplement: S8 Fig — (TIF) [file pone.0324708.s008.tif]
